# Cognition-associated gray matter volume alterations in long-COVID show sex-specific patterns

**DOI:** 10.3389/fpsyt.2025.1653295

**Published:** 2025-10-06

**Authors:** Antonia Toepffer, Marlene Früh, Tonia Rocktäschel, Johanna Ballez, Marie Troll, Daniel Güllmar, Kathrin Finke, Philipp A. Reuken, Andreas Stallmach, Sabine Vonderlind, Ildiko Rita Dunay, Christian Gaser, Martin Walter, Bianca Besteher

**Affiliations:** ^1^ Department of Psychiatry and Psychotherapy, Jena University Hospital, Jena, Germany; ^2^ Medical Physics Group, Institute for Diagnostic and Interventional Radiology, Jena University Hospital, Jena, Germany; ^3^ Department of Neurology, Jena University Hospital, Jena, Germany; ^4^ German Center for Mental Health (DZPG), Jena, Germany; ^5^ Center for Intervention and Research on Adaptive and Maladaptive Brain Circuits Underlying Mental Health (C-I-R-C), site Halle-Jena-Magdeburg, Jena, Germany; ^6^ Department of Internal Medicine IV, Gastroenterology, Hepatology and Infectious Diseases, Jena University Hospital, Jena, Germany; ^7^ Center for Behavioral Brain Sciences (CBBS), Magdeburg, Germany; ^8^ Institute of Inflammation and Neurodegeneration, Otto von Guericke University Magdeburg, Magdeburg, Germany

**Keywords:** long-covid, post-COVID, COVID-19, GMV, VBM, MOCA, sex-difference, cognitive impairment

## Abstract

**Introduction:**

The long-term effects of the coronavirus disease 2019 (COVID-19) are a major concern in today’s society, with cognitive impairment being an important manifestation. Notably, men and women exhibit differences in disease progression and the prevalence of long-COVID. This study aims to investigate sex differences in cognitively impaired long-COVID individuals and their potential association with alterations in gray matter volume (GMV).

**Methods:**

We conducted MRI at 3 Tesla to investigate brain structural correlates of cognitive impairment in long-COVID patients using voxel-based morphometry (VBM) and compared these patients to a healthy control (HC) group (n=30, female=13, male=17). Long-COVID patients underwent scanning and neuropsychiatric assessment on average 9.9 months after their acute and mostly mild COVID-19 infection. Based on Montreal Cognitive Assessment (MoCA) scores, they were classified into two groups: the PCn group, showing preserved cognitive function with MoCA scores of 26 or higher (n=36, female=23, male=13), and the PCcog group, characterized by cognitive impairment with MoCA scores below 26 (n=28, female=15, male=13). Subsequent analyses were performed separately for males and females to investigate sex-specific brain structural correlates of cognitive impairment.

**Results:**

Our analysis revealed significant GMV alterations in long-COVID patients across various brain regions, encompassing both shared and sex-specific regional changes. In females, these alterations were more restricted, affecting anterior frontal, limbic, and diencephalic regions. In males, GMV alterations were more widespread, involving neocortical regions such as the parietal, occipital, and motor cortices, and were characterized by a greater number of affected clusters.

**Discussion:**

Our findings demonstrate GMV alterations in both men and women with cognitive impairment, exhibiting sex-specific differences in affected regions. These differences suggest potentially distinct underlying mechanisms, highlighting the need for further research into their functional implications and relevance for personalized treatment strategies.

## Introduction

The coronavirus disease 2019 (COVID-19) pandemic, caused by severe acute respiratory syndrome coronavirus 2 (SARS−CoV−2) has raised awareness of its long-term effects on human health. While many individuals recover fully, some continue to experience symptoms well beyond the initial infection. This condition is referred to by various terms, including long-COVID, post-COVID-19 syndrome, post-COVID condition (PCC), or post-acute COVID-19 syndrome (PACS). In this study, the term long-COVID is used.

Long-COVID encompasses both ongoing symptomatic COVID-19 (4–12 weeks) and post-COVID-19 condition (12 weeks or more) (1). It can occur in both hospitalized and non-hospitalized individuals and is known to affect multiple organ systems (2). During the early stages of the pandemic cognitive impairment has been reported in 13,5% to 28.85% of individuals with prior SARS-CoV2 infection (3, 4). A large English study conducted between 2020 and 2022 with 112,964 participants found that objectively measurable cognitive deficits persisted for a year or more following SARS-CoV-2 infection, particularly in those with severe illness, prolonged symptoms, or infections during the early phase of the pandemic (5). However, cognitive impairment has also been observed regardless of disease severity (6, 7).

From the onset of the pandemic, sex differences in infection rates and disease progression became evident. Men exhibited higher mortality rates and more severe disease courses (8, 9). A Swedish study on ICU patients conducted between 2020 and 2022 found that critically ill men faced a greater risk of poor long-term outcomes (10), a disparity linked to comorbidities, behavioral and lifestyle factors (9, 11), aging, and biological sex differences (8). While men were more prone to severe acute illness, women appeared to be at higher risk for persistent symptoms (12, 13). A comprehensive review involving 1.32 million patients revealed that women were significantly more likely than men to experience long-term effects of COVID-19 across multiple categories (14, 15). This may be attributed to greater symptom self-awareness in women compared to men (16) as well as a more persistent immune response (17). Beyond these biological and perceptual factors, sex-specific differences in pandemic-related psychosocial stressors and in coping strategies may also contribute to the observed disparities (18).

While previous research has investigated structural brain alterations following SARS-CoV-2 infection using various imaging techniques, findings remain inconsistent. Some studies suggest that COVID-19 can lead to changes in brain structure, including negative associations between gray matter volume (GMV) and neuropsychiatric symptoms, indicative of atrophy and loss of connectivity (19, 20). Others report positive correlations of GMV in specific brain regions and memory loss, a key neuropsychiatric symptom (21), or no alteration at all (22). These positive correlations, likely reflecting ongoing low-grade inflammation in the hippocampus, basal ganglia, thalamus, and insula, were also observed by our group (23). We hypothesized that structural alterations in these regions, which are partly components of the limbic system and the secondary olfactory network, might contribute to the neuropsychiatric symptoms observed in long-COVID (23).

Additionally, we found a negative correlation between functional connectivity in the caudate and the left precentral gyrus and Montreal Cognitive Assessment (MoCA) scores (24).

Given the inconsistent findings regarding long-COVID related brain structural changes and the lack of sex-disaggregated data, further investigation is essential to clarify the potential impact of COVID-19 on brain structure and its relationship to cognitive impairment. Reliable and standardized techniques are necessary to investigate structural brain changes in people with long-COVID, focusing on well characterized subgroups defined by age, sex, clinical features, and recovery time. Voxel-based morphometry (VBM) is a well-established neuroimaging technique for assessing GMV changes in specific brain regions (25) and has been widely applied in the study of structural alterations across various neurological and psychiatric conditions (26).

This study aimed to determine whether cognitive deficits in long-COVID individuals, assessed by the MoCA, are associated with GMV changes compared to a long-COVID group without cognitive deficits and a healthy control (HC) group. Given the previously described sex-related clinical disparities, we also hypothesized sex-specific patterns of GMV alterations.

## 2 Methods

### 2.1 Participants and assessments

A total of 94 participants were included in this cross-sectional case-control study and assigned to one of three groups.

All long-COVID patients were recruited from the long-COVID outpatient clinics of the Department of Internal Medicine IV (Infectiology) and the Department of Neurology at the University Hospital of Jena. Participants were included based solely on a confirmed long-COVID diagnosis to ensure a broad symptom spectrum; without requiring specific symptom profiles. A positive polymerase chain reaction (PCR) test was used to verify SARS-CoV-2 infection at both clinics. The diagnosis of long-COVID was based on the 2021 NICE and AWMF guidelines, which defined long-COVID as symptoms newly occurring after SARS-CoV-2 infection, not explained by other medical conditions, and persisting for > 4 weeks after infection onset (27, 28). The presence and duration of long-COVID symptoms were systematically recorded in a descriptive way via self-report, the symptom spectrum was as multi-facetted as in representative population-based studies with pronounced fatigue and cognitive impairment (29). Accordingly, 61.1% of the PCn cohort and 73.1% of the PCcog cohort reported cognitive impairment, while 75% of the PCn group and 96.2% of the PCcog group reported fatigue. Additional symptoms are listed in **Table 1** (see also **Supplementary Table 2**). On average, the PCn group experienced 5.14 long-COVID symptoms, whereas the PCcog group reported 6.9 (see **Table 1**). Medical history, including information on the timing of previous SARS-CoV-2 infection(s), COVID-vaccination status and the severity of the acute COVID-19 infection as defined by the World Health Organization (WHO), was collected by a certified physician. Severity of COVID-19 was categorized into five levels based on the clinical manifestations and disease progression: uninfected (score 0), ambulatory mild disease (scores 1-3), hospitalized moderate disease (scores 4-5), hospitalized severe disease (scores 6-9), and deceased (score 10) (30). The mean WHO severity among long-COVID participants (both groups) was 2.27 (range 1 to 5, SD 0.89). Patients were enrolled and assessed in the study on average 9.9 months after infection (range 1 to 24.5 months, SD 4).

**Table 1:** Demographic data of the HC, PCn and PCcog groups.

Measures	HC (n=30)	PCn (n=36)	PCcog (n=28)
Demographics			
Age, mean ± SD (years)	42.0 ± 10.8	41.8 ± 11.4	50.04 ± 15
Education, mean ± SD (years)	11.1 ± 1.1	11.2 ± 1	11.07 ± 1.1
Female	13	23	15
Male	17	13	13
Clinical characteristics			
WHO Severity of COVID, mean ± SD	—	2.13 ± 0.67	2.48 ± 1.12
Time since COVID (months), mean ± SD	—	9.9 ± 3.7	9.96 ± 4.39
MoCA mean ± SD	27.97 ± 1.9	27.4 ± 1.5	23.64 ± 1.57
Number of COVID-vaccinations, mean ± SD	1.9 ± 1.24	1.7 ± 0.63	1.8 ± 0.75
Number of long-COVID symptoms, mean (range)	—	5.14 (11)	6.9 (11)
Duration of symptoms (months), mean ± SD	—	10.0 ± 4.3	9.4 ± 5.3
Subjective Symptoms (%)			
Fatigue		75	96.2
Cognitive impairment		61.1	73.1
Shortness of breath		52.8	73.1
Muscle/Joint pain		50	80.8
Sleep disturbance		44.4	61.5
Cough		44.4	69.2
Loss of smell/taste		33.3	30.8
Depressed mood		19.4	46.2
Anxiety		16.7	23.1
Attentional deficits		13.9	7.7
Headache		5.6	80.8
Word retrieval difficulties		5.6	3.8
Palpitations		5.6	0
Neuropathy		2.8	26.9
Hair loss		2.8	3.8
Paresthesia		2.8	0
Emotional stress		2.8	0
Sore throat		2.8	0
Sinusitis		2.8	0
Perceived ocular pressure		2.8	0
Sweating		2.8	0
Cold intolerance		2.8	0
Dizziness		2.8	0
Decreased appetite		2.8	0
Visual impairment		2.8	0
Food intolerance		0	3.8
Tinnitus		0	3.8
Brain fog		0	3.8

HC, healthy control group; PCn, long-COVID group with MoCA≥26; PCcog, long-COVID group with MoCA<26; SD, standard deviation; WHO, World Health Organization; MoCA, Montreal Cognitive Assessment.

Long-COVID individuals were stratified based on their cognitive performance assessed using the MoCA score (31): those with a MoCA score ≥ 26 were assigned to the cognitively unimpaired long-COVID group (PCn; n=36; mean age 41.8; SD 11.4), while participants with a MoCA score < 26 were assigned to the cognitively impaired long-COVID group (PCcog; n= 28; mean age 50.04; SD 15). A healthy control group (HC n= 30; mean age yrs 42.0; SD 10.8) was included for comparison. All participants self-identified as either male or female, and subsequent analyses were stratified accordingly by sex. To enable sex-specific analysis, each group was further subdivided by sex. Female and male participants were labeled f-PCn/m-PCn, f-PCcog/m-PCcog and f-HC/m-HC, respectively. Group distributions were as follows: the cognitively unimpaired long-COVID group (PCn) included 23 female (f-PCn) and 13 male participants (m-PCn); the cognitively impaired long-COVID group (PCcog) comprised 15 female (f-PCcog) and 13 male participants (m-PCcog); and the healthy control group (HC) consisted of 13 female (f-HC) and 17 male participants (m-HC)

The HC group was recruited via announcements of the study in the local newspaper and on social media accounts of the clinic. To ensure that participants in the HC group had not previously been infected with SARS-CoV-2 at the time of assessment, the absence of SARS-CoV-2–specific antibodies was confirmed by serological testing. Serology results were further validated using a Western blot to distinguish between antibodies arising from natural infection and those induced by vaccination.

All participants were screened via telephone to exclude those with past or current psychiatric disorders and current addiction. Additional exclusion criteria included contraindications for magnetic resonance imaging (MRI), diseases of the nervous system, a history of traumatic brain injury or loss of consciousness, unmedicated internal medical conditions and severe cognitive impairment (IQ < 80). To exclude the latter, IQ was estimated using the German Multiple Choice Vocabulary Test B (MWT-B) (32).

The study protocol was approved by the local ethics committee of the Jena University Hospital. All participants gave written informed consent. **Tables 1**, **2** summarize the demographic and psychometric data.

**Table 2:** Sex-stratified clinical and cognitive measures across cohorts.

Measures	Male			Female		
	m-HC M (SD)	m-PCn M (SD)	m-PCcog M (SD)	f-HC M (SD)	f-PCn M (SD)	f-PCcog M (SD)
Time since infection (months)	—	11.1 (2.7)	9.33 (3.8)	—	9.2 (4)	10.47 (4.9)
MoCA	28.24 (1.8)	27.2 (1.4)	23.54 (1.7)	27.6 (2.1)	27.5 (1.5)	23.73 (1.5)
WHO-Severity	—	2.33 (1)	2.2 (1)	—	2.05 (0.5)	2.73 (1.2)

M, mean; SD, standard deviation; HC, healthy control group, PCn, long-COVID group with MoCA≥26; PCcog, long-COVID group with MoCA<26.

### 2.2 Magnetic resonance imaging

Each participant underwent high-resolution T1-weighted MRI scans using a standard quadrature head coil and an axial 3-dimensional magnetization-prepared rapid gradient echo (MP-RAGE) sequence (TR 2400 ms, TE 2.22 ms, α 8°, 208 contiguous sagittal slices, FoV 256 mm, voxel resolution 0.8 x 0.8 x 0.8 mm, acquisition time 6:38 min) on a 3 Tesla Siemens Prisma fit (Siemens, Erlangen, Germany). All scans were checked for imaging artefacts.

### 2.3 Voxel-based morphometry

VBM analysis was performed using the CAT12 (Computational Anatomy Toolbox 12) developed by the Structural Brain Mapping group at University Hospital Jena, Germany, and implemented in SPM12 (Statistical Parametric Mapping, Institute of Neurology, London, UK). The T1-weighted images underwent bias-field correction to account for field homogeneity, followed by spatial normalization using the DARTEL algorithm (33). The images were segmented into white matter, gray matter and cerebrospinal fluid (34). To improve the accuracy of the segmentation process, it was extended to account for partial volume effects (35). Adaptive maximum a posteriori estimation was applied, and a hidden Markov random field model was used. To exclude artefacts at the grey-white matter boundary, an internal grey matter threshold of 0.1 was applied. After pre-processing, all scans were subjected to an automated quality control protocol. As CAT12 does not apply a fixed motion-exclusion threshold; instead, we used its image quality ratings and visual inspection to identify and exclude scans affected by motion artifacts. 2 participants from the long- COVID patient group had to be excluded from further analysis due to poor image quality at that point. The remaining images were smoothed with an 8 mm FWHM Gaussian kernel, which represents a widely used compromise between sensitivity and anatomical specificity, satisfies assumptions of Gaussian random field theory for voxel-based inference, and facilitates comparability with prior morphometry studies (36, 37).

### 2.4 Statistics

The statistical analysis was performed using the general linear model approach, implemented in SPM12. Groups were compared using two-sample t-tests. To account for associated variance, we included total intracranial volume (TIV), age and sex as confounding variables in the VBM analysis. As a non-parametric statistic, we applied threshold-free cluster enhancement (TFCE) with 5000 permutations (38) to all analyses and corrected for multiple comparisons via the family wise error method (FWE) at p<0.05. For atlas labelling of significant clusters we used the Neuromorphometrics Atlas (http://www.neuromorphometrics.com). We first performed this analysis for the overall groups of healthy controls (HC), long-COVID patients without cognitive symptoms according to MoCA (PCn) and long-COVID patients with cognitive impairment (PCcog). In a second step we performed these analyses separately for female and male participants, adding TIV and age as confounding variables. Complementary statistical analysis of clinical and demographic data was conducted in IBM SPSS Statistics (version 29 0.2.0.). A significance level of p≤ 0.05 was applied. The chi-square test assessed sex distribution among groups. Kruskal-Wallis tests were used to compare age, years of education and TIV. Mann-Whitney U tests examined differences in WHO severity of acute COVID-19 infection and time since infection between PCn, f-PCn, m-PCn and PCcog. f-PCcog, m-PCcog. ANCOVA was performed to assess the influence of covariates on MoCA scores.

## 3 Results

### 3.1 Clinical and demographic results

#### 3.1.1 Overall analysis

No significant difference in sex distribution was observed between the HC, PCn and PCcog groups (χ2 (2)=2.793, p=0.247; Chi-square test). Similarly, there were no significant differences in years of education (H (2)=0.153, p=0.926) or TIV (n=92, H (2)=0,266, p=0.875; Kruskal-Wallis test). A significant group difference in age was found (H (2)=7.061, p=0.029; Kruskal-Wallis test), with PCcog participants being significantly older than those in the HC (U = 280.0, Z=-2.180, p=0.029) and PCn groups (U = 323.0, Z=-2.451, p=0.014; Mann-Whitney U test). No significant differences between the PCn and PCcog groups in WHO severity scores (U = 276.0, Z=-1.401, p=0.161) and time since infection (U = 455,5, Z=-0.242, p=0.809; Mann-Whitney U test) were found.

As expected, due to group definitions based on cognitive status, MoCA scores differed significantly between the PCcog group and the HC (U = 41.500, Z=-5.963, p=<0.001), and the PCn groups (U = 0.000, Z=-6.900, p=<0.001), while the HC and PCn groups did not differ significantly (U = 406.000, Z=-1.759, p= 0.079; Mann-Whitney U test). ANCOVA results showed that TIV, age, years of education, time since infection, and WHO severity were not significantly associated with MoCA performance. Only group affiliation, used to define cognitive status, had a significant effect on MoCA score (see **Table 3**).

**Table 3:** overall ANCOVA.

ANCOVA- overall				
Cohorts	Measures	F	Partial eta squared	P-value
HC (n=30), PCn (n=34), PCcog (n=25)	TIV	.071	.001	.791
	Age	2.168	.026	.145
	Education	.139	.002	.711
	Subgroup	41.695	.510	<.001
	Sex	.000	.000	.995
	Sex*subgroup	.1.172	.028	.315
PCn (n=31), PCcog (n=21)	WHO severity	.279	,027	.890
	Time since infection	0.752	.018	.391
	Subgroup	14.081	.260	<.001
	Sex	1.374	.033	.248
	Subgroup*sex	.364	.009	.550

n = number of participants included in the analysis for each dependent variable; for TIV, two participants were excluded due to insufficient MRI image quality.

#### 3.1.2 Sex-stratified analysis

In both female and male subgroups, no significant differences were observed between f-PCn and f-PCcog and m-PCn and m-PCcog in terms of WHO severity (females: U = 79.0, Z=-2.385, p=0.114, males: U = 40.5, Z=-0.579, p=0.720) or time since infection (females: U = 158.5, Z=-0.419, p=0.680, males: U = 58.5 Z=-0.781, p=0.443; Mann-Whitney U test).

Among women, no significant group differences in years of education (H (2)=0.353, p=0,838), TIV (n=92, H (2)=0,459, p=0,795) or age (H (2)=2,004, p=0,367; Kruskal-Wallis test) were found. Similarly, no significant differences were found among men for education (H (2)=0,192, p=0,908), TIV (n=92, H (2)=0,610, p=0,737) or age (H (2)=5,421, p=0,066; Kruskal-Wallis test). Separate ANCOVAs for women and men confirmed that none of the potential confounding variables significantly influenced MoCA performance (see **Table 4**). As expected, group classification significantly affected MoCA scores in both subgroups, consistent with the criteria used for defining cognitive status.

**Table 4:** Sex-stratified ANCOVA.

ANCOVA (women)				
Cohorts	Measures	F	Partial eta squared	P-value
f-HC (n=13), f-PCn (n=23), f-PCcog (n=14)	TIV	.353	.008	.555
	Age	3.263	.069	.078
	Education	.438	.010	.512
	Subgroup	20.413	.481	<.001
f-PCn (n=22), f-PCcog (n=11)	WHO severity	.634	.089	.643
	Time since infection	.441	.017	.512
	Subgroup	27.903	.518	<0.001
ANCOVA (Men)				
m-HC (n=17), m-PCn (n=11), m-PCcog (n=11)	TIV	.080	.002	.779
	Age	.037	.001	.849
	Education	.000	.000	.996
	Subgroup	22.728	.579	<0.001
m-PCn (n=9), m-PCcog (n=10)	WHO severity	.046	.007	.955
	Time since infection	.426	.032	.525
	Subgroup	7.164	.355	.019

HC, healthy control group; f-PCn, female long-COVID group with MoCA≥26; f-Ccog, female long-COVID group with MoCA<26; m-PCn, male long-COVID group with MoCA≥26; m-Pcog, male long-COVID group with MoCA<26; TIV, total intracranial volume.

### 3.2 Imaging results

Overall analyses and sex-separated analyses of the HC, PCn and PCcog groups revealed several significant clusters (p<0.05, FWE-corrected) showing GMV alterations between the HC and both PC groups.

#### 3.2.1 Overall comparison

Significant clusters exceeding a size threshold of k_E_>100 were identified for each comparison and are displayed in **Supplementary Table 1** and **Supplementary Figure 1**.

#### 3.2.2 Sex-stratified analysis

In women, no significant clusters of significant GMV differences were found for the comparisons f-PCn<f-PCcog and f-PCn>f-PCcog, whereas the remaining comparisons revealed significant clusters. In men, no significant clusters emerged for the contrasts m-HC<m-PCcog, m-HC>m-PCn, m-PCn<m-PCcog; all other comparisons yielded significant results.

**Figure 1:**
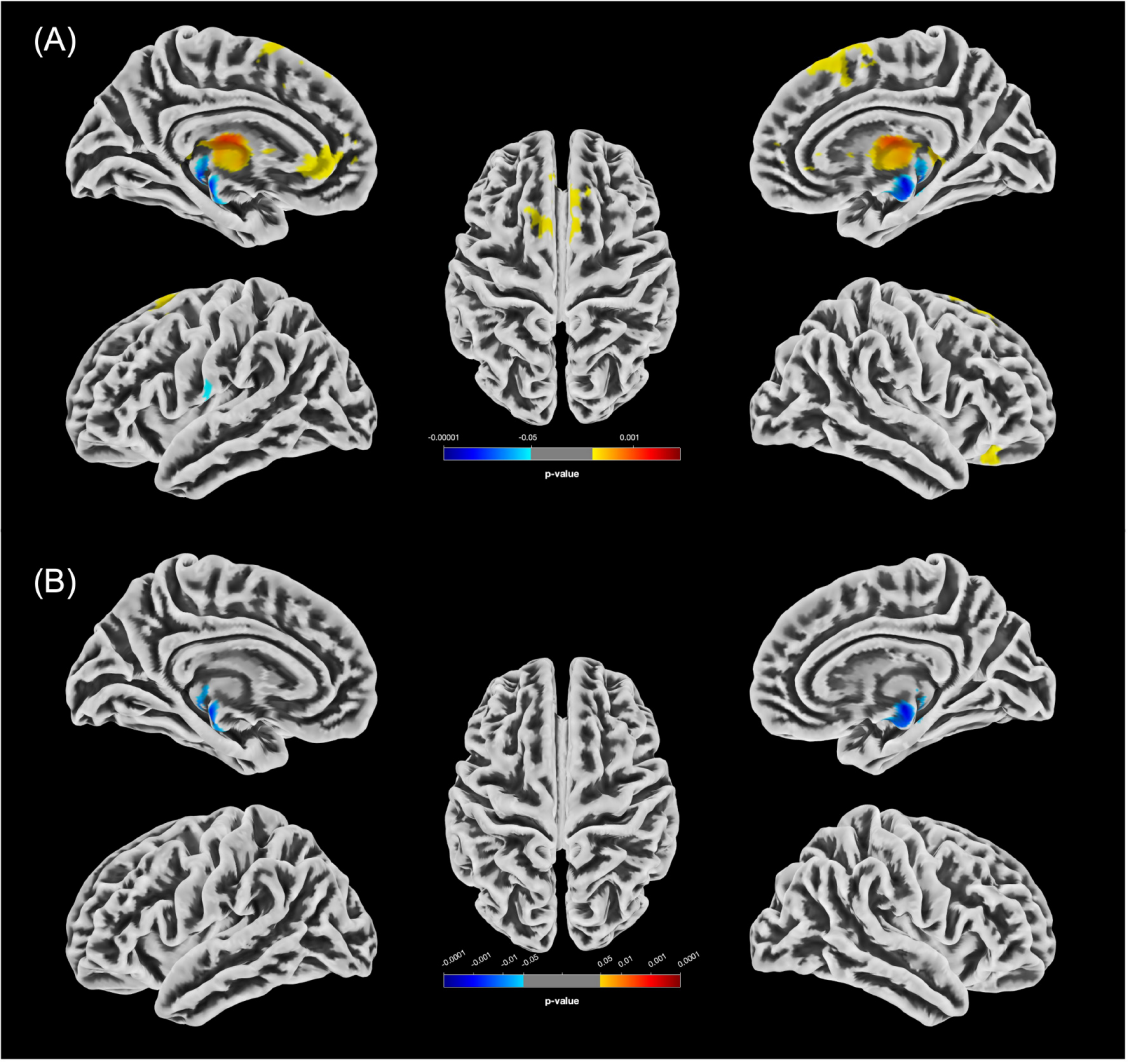
Statistically significant GMV differences between the female groups (p<0.05, FWE-corrected). **(A)** Increases in GMV in f-PCn relative to f-HC (f-HC<f-PCn) are shown in yellow/red; decreases (f-HC>f-PCn) in blue. **(B)** Significant clusters of reduced GMV in f-PCcog compared to f-HC are displayed in blue.

**Figure 2:**
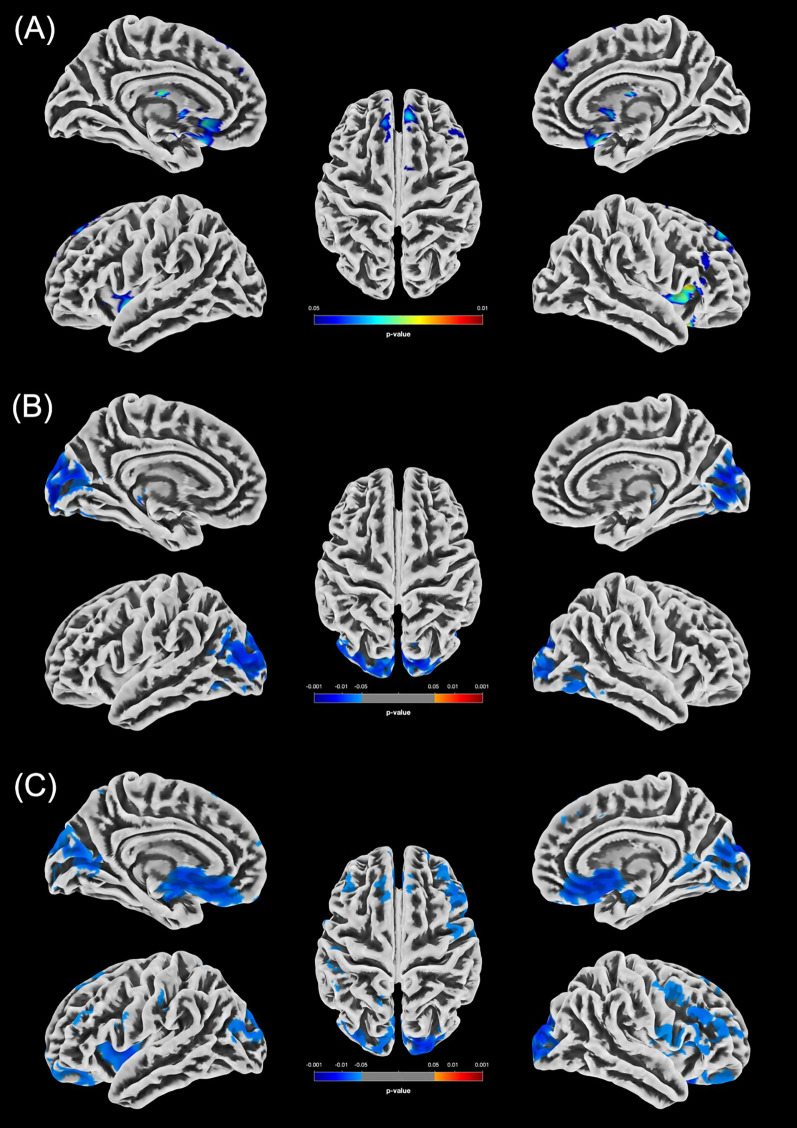
Statistically significant GMV differences between the male groups (p < 0.05, FWE-corrected). **(A)** Increased GMV in the m-PCn group compared to m-HC (m-HC < m-PCn) is displayed in blue/green. **(B)** Reduced GMV in m-PCcog compared to m-HC is displayed in blue. **(C)** Increased GMV in the m-PCn group compared to the m-PCcog group (m-PCn > m-PCcog) is displayed in blue.

An overview of all significant clusters (k_E_>100) is provided in **Table 5**, with their spatial distribution illustrated in **Figure 1** (women) and **Figure 2** (men).

**Table 5:** Sex-stratified GMV differences between long-COVID cohorts and controls.

Contrast	H	K	Overlap region	P-value	TFCE	Peak cluster (x,y,z)
f-HC<f-PCcog	L	106	Thalamus Caudate	.031	976	-10,-12,20
m-HC<m-PCcog						
f-HC>f-PCcog	L	345	Ventral Diencephalon Thalamus	<.001	4118	-2,-10,-10
	R					
	L	374	Ventral Diencephalon Thalamus Hippocampus Parahippocampal gyrus	.007	1823	-20,-24,-8
m-HC>m-PCcog	L	366	Ventral Diencephalon Hippocampus Thalamus Parahippocampal gyrus	.006	1240	-20,-22,-9
	R	14454	Superior occipital gyrus Cuneus Occipital pole	.007	1208	16,-88,21
f-HC<f-PCn	L	7006	Thalamus Caudate	<.001	2550	-9,-10,15
	L	1891	Superior frontal gyrus Supplementary Motor Cortex	.03	603	-14,8,72
	R	277	Medial Orbital gyrus Posterior orbital gyrus	.031	599	21,32,-27
	L	856	Superior frontal gyrus medial segment Anterior cingulate gyrus Medial Frontal cortex	.033	585	-3,54,6
	R		Superior frontal gyrus medial segment Anterior cingulate gyrus			
m-HC<m-PCn	L	15320	Putamen Caudate Accumbens Area Medial orbital gyrus Subcallosal area Gyrus rectus	.003	2716	-16,18,-10
	R	675	Superior frontal gyrus Superior frontal gyrus medial segment	.027	1654	9,51,42
	L	742	Superior frontal gyrus Superior frontal gyrus medial segment	.031	1591	-10,36,48
f-HC>f-PCn	L	336	Ventral Diencephalon Thalamus	<.001	4023	-2,-10,-10
	R					
	L	612	Ventral Diencephalon Thalamus Hippocampus Parahippocampal gyrus	<.001	2451	-20,-24,-8
m-HC>m-PCn						
f-PCn>f-PCcog						
m-PCn>m-PCcog	L	26978	Putamen Medial orbital gyrus Accumbens Area Caudate Subcallosal area Gyrus rectus Posterior orbital gyrus Anterior insula	.008	1889	-16,16,-12
	R	13113	Superior occipital gyrus Occipital pole Cuneus	.009	1791	18,-90,26
	L	228	Superior frontal gyrus Supplementary Motor Cortex Superior frontal gyrus medial segment	.043	1050	-12,26,64
	L	315	Supramarginal gyrus Postcentral gyrus Parietal operculum	.043	1049	-56,-28,32
	L	359	Superior frontal gyrus Superior frontal gyrus medial segment Supplementary Motor Cortex	.043	1041	-12,33,51
	R	329	Superior frontal gyrus medial segment Superior frontal gyrus	.045	1025	10,40,42
	L	371	Middle frontal gyrus	.046	1019	-42,33,30
	L	117	Superior parietal lobule Precuneus	.046	1013	-16,-60,62
	L	183	Precentral gyrus Opercular part of the inferior frontal	.047	1004	-58,9,21

H, hemisphere; k, cluster size; TFCE, Threshold-free Cluster Enhancement.

## 4 Discussion

Our analyses revealed significant GMV alterations across multiple brain regions, comprising both shared and sex-specific patterns.

### 4.1 Common GMV alterations in male and female participants

In the HC>PCcog comparison, several brain regions showed overlapping GMV reductions in both sexes, indicating shared structural alterations. Both m-PCcog and f-PCcog participants exhibited GMV reductions in the left ventral diencephalon, thalamus, hippocampus, and parahippocampal gyrus. Among these, the thalamus and hippocampus are particularly critical for cognitive function (39, 40). Men additionally showed alterations in right occipital areas. These findings are consistent with previous findings on GMV alterations associated with long-COVID. Diez-Cirarda et al. reported GMV reductions in limbic areas, among others, associated with cognitive dysfunction (41). Similarly, a UK Biobank study investigating brain changes in 401 participants over a long-term follow-up period found reductions in gray matter thickness and tissue contrast in the parahippocampal gyrus and orbitofrontal cortex, along with an overall greater progression of cognitive decline in COVID-19 patients (20).

The hippocampus has likewise emerged as a region of concern in individuals recovering from SARS-CoV-2 infection. As a key structure for cognition and particularly episodic memory (42), it is critically implicated in the pathophysiology of neurodegenerative disorders, such as Alzheimer`s disease, and psychiatric conditions including major depressive disorder (43–45). In the context of long-COVID, hippocampal structural and functional alterations, potentially affecting adult neurogenesis, have been linked to memory loss and an accelerated progression of neurodegenerative processes (46, 47).

In the present study, long-COVID patients exhibited consistently reduced hippocampal GMV compared to HC, in line with findings from Capelli et al., Kamasak et al. and Invernizzi et al. (48–50). However, contrasting results have been reported in two large-scale studies that found increased hippocampal GMV (51), with Lu et al. additionally describing a positive correlation with memory impairment (Lu et al., 2020). These divergent findings underscore the complexity of structural brain alterations associated with long-COVID.

The coexistence of both increases and decreases in GMV of different brain regions may reflect a dynamic interplay between neurodegenerative processes and compensatory mechanisms, such as neuroplasticity (52, 53). Neuroinflammation, a key focus of long-COVID research, is thought to contribute to structural brain changes through cytokine-mediated disruption of the blood-brain barrier, neurovascular damage, and impaired neurogenesis (54–62). Evidence from previous coronavirus outbreaks (SARS, MERS) supports this mechanism (63, 64). These pro-inflammatory responses resemble those implicated in cancer therapy-related cognitive impairment, suggesting shared pathophysiological pathways (65, 66). Under certain conditions, however, microglial activation may promote neurogenesis, depending on cytokine profiles and concentrations (67). This dual role could help explain the heterogeneous pattern of GMV alterations observed in long-COVID.

Beyond hippocampal and cortical regions, the amygdala has been implicated in COVID-19 related neurocognitive changes (50, 68). Invernizzi et al. reported structural and functional alterations not only in the hippocampus but also the left amygdala, where reduced connectivity was shown to specifically mediate spatial working memory deficits (50). A cross-sectional study involving 75 individuals, including COVID-19 survivors with and without brain fog and healthy controls, found that both COVID-19 groups showed reduced gray matter concentrations in the left inferior temporal gyrus, left fusiform gyrus, and right orbital gyri compared to healthy controls. In addition, participants with brain fog exhibited further reductions in the bilateral caudate nuclei, right putamen/pallidum, and amygdala (68).

This highlights the role of limbic circuitry in cognitive sequalae following SARS-CoV-2 infection. Complementary longitudinal work in healthy individuals without prior SARS-CoV-2 infection linked transient volumetric increases in the amygdalae to stress- and anxiety-related processes following the COVID-19 outbreak and lockdown, with GMV gradually decreasing over time after lockdown relief (69). In our study, however, the amygdala did not emerge as a region of interest. Nevertheless, other limbic structures showed relevant alterations.

Although the overall analysis was secondary to the sex-stratified results, it revealed several regions of interest relevant to cognitive functioning, including the hippocampus, entorhinal area, posterior cingulate gyrus, angular gyrus, and planum temporale (see **Supplementary Table 1** and **Supplementary Figure 1**).

### 4.2 Sex-specific GMV alterations

#### 4.2.1 GMV alterations observed in female participants

The distribution of clusters with altered GMV in female participants compared to males in our analysis was more restricted, predominantly involving anterior frontal areas as well as limbic and diencephalic regions, including the ventral diencephalon, hippocampus and thalamus. No significant differences were found between the f-PCn and f-PCcog groups. Notably, the left thalamus consistently demonstrated GMV alterations across all statistically significant female subgroup comparisons, with both increases and decreases observed. Given its heterogenous structure and its central role in cognitive processes (70), thalamic involvement may be particularly relevant to neuropsychiatric manifestations of long-COVID. Supporting this, VBM in patients with mild cognitive impairment, unrelated to COVID-19, similarly revealed volumetric reductions in the left thalamus, along with alterations in the hippocampus and amygdala (39). This convergence underscores the thalamus as a central node whose vulnerability may extend across different conditions associated with cognitive decline.

GMV alterations specific to female participants were also detected in the anterior cingulate gyrus and medial frontal cortex.

#### 4.2.2 GMV alterations observed in male participants

In men, the distribution of statistically significant GMV clusters was broader than in women, extending into parietal, occipital and motor areas. Additionally, the number of clusters was greater compared to women. A consistent pattern of reduced GMV emerged in the m-PCcog group, with the occipital pole, cuneus, and superior occipital gyrus repeatedly showing GMV reductions. While the occipital pole and superior occipital gyrus are not primarily associated with cognitive functions, the cuneus plays a role in working memory, which is crucial for performance in complex cognitive tasks (71, 72) and is often implicated in early stages of neurodegenerative and psychiatric conditions (73, 74). In addition to the previously mentioned regions, the putamen exhibited notable GMV increases in the m-PCn group compared to both the m-HC and the m-PCcog groups, reinforcing the notion of sex-specific structural alterations in long-COVID. The putamen, a key component of the basal ganglia along with the caudate nucleus and pallidum (75), plays a central role in motor control, learning, behavior regulation, and emotional processing (76) and has increasingly been implicated in the context of long-COVID. A systematic review highlighted the frontal, temporal, and parietal lobes, as well as the cerebellum, hippocampus, amygdala, and basal ganglia as key regions affected in post-COVID conditions (77). In line with this, Vakani et al. found that persistent COVID-19 symptoms were significantly associated with smaller putamen volume, impaired cognitive performance and poorer mental health and sleep quality (78). Heine et al. reported shape deformations and decreased GMV in the left thalamus, putamen and pallidum in post-COVID fatigue patients (79). These findings converge with our results and underscore the relevance of basal ganglia alterations in long-COVID. Moreover, recent work from our group linked changes in corticostriatal connectivity to cognitive impairment in long-COVID patients (24), potentially mediated by ACE2 receptor expression in the basal ganglia, which facilitates SARS-CoV-2 entry (24).

The broader distribution of GMV alterations observed in cognitively impaired men compared to women may be linked to sex-specific immune response patterns. Men are more prone to excessive inflammatory responses, including cytokine storms, which are associated with poor COVID-19 outcomes and may contribute to neural damage (8). However, since our cohort primarily included individuals with mild disease courses, this mechanism alone is unlikely to fully account for the observed sex-differences, particularly considering the absence of significant GMV alterations between m-HC and m-PCn participants. Instead, the findings likely reflect underlying biological factors such as hormonal influences and immune regulatory differences. Females generally exhibit stronger innate immune responses, greater resistance to viral infections, and lower levels of inflammatory mediators (8, 16, 80, 81), potentially mitigating neuroinflammation and limiting GMV changes in long-COVID. Importantly, the differing spatial distribution of GMV alterations between sexes was not accompanied by measurable differences in cognitive performance, as indicated by comparable MoCA scores across male and female participants.

Direct comparisons with prior work are limited, as few studies have examined sex-specific structural brain changes in long-COVID. One VBM study in men reported right hippocampal volume reductions shortly after Omicron infection, but was limited by the absence of a control group, small sample size, and the fact that cognitive impairment was not addressed in the study (82).

In summary, our findings demonstrate sex-specific GMV alterations in individuals with long-COVID, with men showing a broader distribution of affected regions despite the higher reported prevalence of long-COVID in women. Both sexes exhibited changes in brain areas relevant to cognition, with notable overlap between groups. However, given the cross-sectional design and limited sample size, the generalizability and temporal stability of these findings remain uncertain. Furthermore, the MoCA may have limited sensitivity in younger participants, potentially affecting the accuracy of cognitive assessment (83). Longitudinal, sex-stratified studies are needed to clarify the long-term neuropsychiatric effects of SARS-CoV-2. Also, our future work will focus on extending our analyses to larger and more heterogeneous samples through national and international collaborations, thereby improving the generalizability and robustness of our findings.

## Data Availability

The datasets presented in this article are not readily available because informed consent and ethics approval for anonymous public use were not obtained and cannot be secured retrospectively. Pseudonymized data may be provided upon reasonable request. Requests to access the datasets should be directed to Bianca Besteher (bianca.besteher@med.uni-jena.de). All image and statistical analyses were conducted using the openly available CAT12 toolbox and IBM SPSS Statistics; no custom code beyond these packages was developed.
