# Supplementary material for: Cognition-associated gray matter volume alterations in long-COVID show sex-specific patterns
Source: Front Psychiatry. 2025 Oct 6;16:1653295. doi: 10.3389/fpsyt.2025.1653295 (PMC12536713; doi:10.3389/fpsyt.2025.1653295)
Supplement: Supplementary file 1 [file DataSheet1.docx]

**Supplementary Figure 1** Statistically significant GMV differences of the overall analysis (p<0.05, FWE-corrected).

(A) Regions with increased GMV in PCn (HC < PCn) are shown in orange/yellow; regions with decreased GMV (HC > PCn) are shown in blue.

(B) Areas with increased GMV in PCcog compared to HC (HC < PCcog) are shown in yellow/red; areas with decreased GMV (HC > PCcog) are shown in blue.

(C) Regions with increased GMV in PCcog (PCn < PCcog) are shown in orange; regions with decreased GMV (PCn > PCcog) are shown in blue.
