# Supplementary material for: Cognition-associated gray matter volume alterations in long-COVID show sex-specific patterns
Source: Front Psychiatry. 2025 Oct 6;16:1653295. doi: 10.3389/fpsyt.2025.1653295 (PMC12536713; doi:10.3389/fpsyt.2025.1653295)
Supplement: Supplementary file 2 [file DataSheet2.docx]

Supplementary Material

**Supplementary Table 1** GMV differences between long-COVID cohorts and controls in the overall analysis

| **contrast** | **Hemi**  **sphere** | **Cluster size** | **Overlap region** | **p-Value** | **TFCE** | **Peak Cluster (x,y,z)** |
| --- | --- | --- | --- | --- | --- | --- |
| HC<PCcog | Left | 18465 | Thalamus Proper  Lateral Ventricle  Caudate | 0.002 | 1976.44 | -9,-12,18 |
|  | Left | 4923 | Cerebellum Exterior | 0.027 | 425.46 | -26,-74,-62 |
|  | Left | 342 | Posterior orbital gyrus  Temporal pole | 0.041 | 272.48 | -36,24,-27 |
|  | Left | 299 | Inferior temporal gyrus  Fusiform gyrus  Temporal pole  Entorhinal area  Parahippocampal gyrus | 0.047 | 230.82 | -30,-3,-46 |
| HC<PCn | Left | 4586 | Thalamus Proper  Lateral Ventricle  Caudate | <0.001 | 1936.66 | -9,-12,16 |
| HC>PCcog | Left | 814 | Ventral DC  Thalamus Proper  Hippocampus  Parahippocampal gyrus | 0.001 | 2556.65 | -20,-24,-8 |
|  | Left  Right | 384 | Ventral DC  Thalamus Proper  Ventral DC  Thalamus Proper | 0.002 | 2177.20 | -2,-10,-9 |
|  | Right  Left | 8207 | Cuneus  Calcarine Cortex  Occipital pole  Superior occipital gyrus  Cuneus | 0.018 | 997.31 | 10,-88,16 |
| HC>PCn | Left | 93013 | Ventral DC  Thalamus Proper  Hippocampus  Parahippocampal gyrus | <0.001 | 3670.99 | -20,-24,-8 |
|  | Right | 351 | Cerebellum Exterior | 0.046 | 200.13 | 45,-64,-50 |
|  | Left | 125 | Cerebellum Exterior | 0.046 | 199.82 | -50,-66,-48 |
|  | Right | 164 | Occipital pole  Inferior occipital gyrus  Superior occipital gyrus | 0.047 | 193.94 | 28,-99,4 |
| PCn<PCcog | Right | 8680 | Middle temporal gyrus  Superior temporal gyrus  Planum polare | 0.007 | 817.93 | 48,-15,-14 |
|  | Right | 3784 | Cerebellum Exterior | 0.013 | 631.66 | 45,-66,-51 |
|  | Left | 9257 | Superior temporal gyrus  Middle temporal gyrus  Transverse temporal gyrus  Planum temporale  Planum polare | 0.016 | 569.21 | -54,-22,0 |
|  | Left | 4476 | Cerebellum Exterior | 0.026 | 451.02 | -40,-63,-62 |
|  | Left | 1396 | Posterior cingulate gyrus  Hippocampus  Lingual gyrus  Precuneus  Thalamus proper | 0.033 | 392.52 | -18,-45,4 |
|  | Left | 1448 | Middle temporal gyrus  Superior temporal gyrus | 0.035 | 377.78 | -57,-54,4 |
|  | Left | 327 | Middle frontal gyrus  Superior frontal gyrus | 0.038 | 356.80 | -28,36,46 |
|  | Right | 1277 | Supramarginal gyrus  Angular gyrus  Parietal operculum | 0.039 | 351.48 | 56,-39,40 |
|  | Left | 203 | Temporal pole  Inferior temporal gyrus | 0.043 | 326.79 | -38,6,-51 |
| PCn>PCcog | Left | 825 | Superior frontal gyrus  Supplementary motor cortex  Superior frontal gyrus medial segment | 0.022 | 334.45 | -14,26,63 |
|  | Left | 191 | Superior occipital gyrus  Occipital pole | 0.030 | 294.36 | -20,-99,27 |
|  | Right  Left | 301 | Cuneus  Calcarine cortex  Cuneus | 0.038 | 261.31 | 9,-84,16 |
|  | Left | 161 | Middle occipital gyrus  Superior occipital gyrus  Occipital pole | 0.042 | 247.84 | -36,-94,18 |

**Supplementary Table 2** Long-COVID Symptomatology and Vaccination Details of PCn and PCcog Participants

|  | **Cohort (MoCA)** | **sex** | **Age (years)** | **Symptom onset** | **Duration of long-COVID symptoms (months)** | **Number of**  **COVID- vaccinations** | **Date**  **and type of vaccination** | **long-COVID symptoms** |
| --- | --- | --- | --- | --- | --- | --- | --- | --- |
| P01 | PCn (27) | m | 60 | 02.06.2020 | *11 | n.a. | n.a. | \| Cognitive/Neurological (attentional deficits); Respiratory/Cardiac (Shortness of breath) \| \| --- \| |
| P02 | PCn (27) | f | 41 | 02.04.2020 | *13 | n.a. | n.a. | General/Systemic (Fatigue, Hair loss, Headache, Muscle/joint pain); Psychiatric/Neuropsychiatric (Anxiety, Sleep disturbance); Cognitive/Neurological (Cognitive impairment, Paresthesia); Respiratory/Cardiac (Cough, Shortness of breath); Sensory (Loss of smell/taste) |
| P03 | PCn (29) | f | 53 | 02.03.2021 | *2 | n.a. | n.a. | \| General/Systemic (Fatigue, Headache, Muscle/joint pain); Psychiatric/Neuropsychiatric (Sleep disturbance); Cognitive/Neurological (Cognitive impairment); Sensory (Loss of smell/taste) \| \| --- \|  \|  \| \| --- \| |
| P04 | PCn (26) | f | 44 | 02/2021 | *2,5 | n.a. | n.a. | \| General/Systemic (Fatigue, Headache, Muscle/joint pain); Psychiatric/Neuropsychiatric (Sleep disturbance); Cognitive/Neurological (Cognitive impairment); Sensory (Loss of smell/taste) \| \| --- \|  \|  \| \| --- \| |
| P05 | PCcog (25) | m | 68 | 12.10.2020 | *7 | n.a. | n.a. | \| General/Systemic (Fatigue, Headache, Muscle/joint pain); Respiratory/Cardiac (Cough, Shortness of breath) \| \| --- \|  \|  \| \| --- \| |
| P06 | PCcog (24) | f | 67 | 03/2020 | *13,5 | n.a. | n.a. | \| General/Systemic (Fatigue, Headache, Muscle/joint pain) \| \| --- \|  \|  \| \| --- \| |
| P07 | PCcog (25) | m | 46 | 03.12.2020 | *5,5 | n.a. | n.a. | \| General/Systemic (Fatigue, Headache, Muscle/joint pain); Psychiatric/Neuropsychiatric (Depressed mood) \| \| --- \|  \|  \| \| --- \| |
| P08 | PCcog (23) | m | 59 | 01/2021 | *4 | n.a. | n.a. | \| General/Systemic (Fatigue); Psychiatric/Neuropsychiatric (Depressed mood, Sleep disturbance); Cognitive/Neurological (Cognitive impairment); Respiratory/Cardiac (Cough, Shortness of breath) \| \| --- \|  \|  \| \| --- \| |
| P09 | PCcog (23) | f | 54 | 29.10.2020 | *6,5 | n.a. | n.a. | \| General/Systemic (Fatigue, Hair loss, Headache, Muscle/joint pain); Psychiatric/Neuropsychiatric (Depressed mood, Anxiety, Sleep disturbance); Cognitive/Neurological (Cognitive impairment); Respiratory/Cardiac (Cough, Shortness of breath); Sensory (Loss of smell/taste) \| \| --- \|  \|  \| \| --- \| |
| P10 | PCn (26) | f | 31 | 12/2020 | *5 | n.a. | n.a. | \| General/Systemic (Fatigue); Cognitive/Neurological (Cognitive impairment, Word retrieval difficulties); Respiratory/Cardiac (Palpitations) \| \| --- \|  \|  \| \| --- \| |
| P11 | PCcog (25) | m | 62 | 05/2020 | *12,5 | n.a. | n.a. | \| General/Systemic (Fatigue); Psychiatric/Neuropsychiatric (Sleep disturbance); Respiratory/Cardiac (Cough, Shortness of breath) \| \| --- \|  \|  \| \| --- \| |
| P12 | PCcog (23) | f | 52 | 29.12.2020 | 1 | n.a. | n.a. | \| General/Systemic (Fatigue, Headache, Muscle/joint pain); Psychiatric/Neuropsychiatric (Depressed mood, Sleep disturbance); Cognitive/Neurological (Cognitive impairment); Respiratory/Cardiac (Cough, Shortness of breath); Sensory (Loss of smell/taste); Other (Food intolerance) \| \| --- \|  \|  \| \| --- \| |
| P13 | PCn (26) | f | 50 | 04/2020 | *13,5 | n.a. | n.a. | \| General/Systemic (Fatigue, Headache, Muscle/joint pain); Psychiatric/Neuropsychiatric (Sleep disturbance, Emotional stress) \| \| --- \|  \|  \| \| --- \| |
| P14 | PCcog (24) | m | 51 | 02/2021 | 3 | n.a. | n.a. | \| General/Systemic (Headache, Muscle/joint pain); Respiratory/Cardiac (Cough, Shortness of breath) \| \| --- \|  \|  \| \| --- \| |
| P15 | PCn (30) | f | 47 | 09.12.2020 | 1 | n.a. | n.a. | \| General/Systemic (Fatigue, Headache); Psychiatric/Neuropsychiatric (Depressed mood, Anxiety, Sleep disturbance); Respiratory/Cardiac (Shortness of breath) \| \| --- \|  \|  \| \| --- \| |
| P16 | PCn (30) | m | 47 | 24.04.2020 | 10 | n.a. | n.a. | \| Psychiatric/Neuropsychiatric (Sleep disturbance); Cognitive/Neurological (Cognitive impairment); Respiratory/Cardiac (Cough, Shortness of breath); Sensory (Loss of smell/taste) \| \| --- \|  \|  \| \| --- \| |
| P17 | PCn (26) | f | 55 | 25.11.2020 | *6 | n.a. | n.a. | \| General/Systemic (Fatigue, Headache, Muscle/joint pain, Sore throat); Psychiatric/Neuropsychiatric (Depressed mood, Anxiety, Sleep disturbance); Cognitive/Neurological (Cognitive impairment); Respiratory/Cardiac (Cough, Shortness of breath) \| \| --- \|  \|  \| \| --- \| |
| P18 | PCn (30) | f | 54 | 26.11.2020 | *6,5 | n.a. | n.a. | \| General/Systemic (Fatigue, Headache, Muscle/joint pain); Cognitive/Neurological (Cognitive impairment); Respiratory/Cardiac (Cough, Shortness of breath); Other (Sinusitis, Perceived ocular pressure) \| \| --- \|  \|  \| \| --- \| |
| P19 | PCn (27) | f | 53 | 23.01.2021 | *5 | 1 | BioNTech 17.05.2021 | \| Sensory (Loss of smell/taste) \| \| --- \|  \|  \| \| --- \| |
| P20 | PCcog (20) | m | 52 | 21.12.2020 | *6 | 2 | 31.03.21, 21.04.21 | \| General/Systemic (Fatigue, Headache, Muscle/joint pain); Psychiatric/Neuropsychiatric (Depressed mood, Anxiety, Sleep disturbance); Cognitive/Neurological (Cognitive impairment, Neuropathy); Respiratory/Cardiac (Cough, Shortness of breath) \| \| --- \|  \|  \| \| --- \| |
| P21 | PCn (26) | m | 50 | n.a. | n.a. | n.a. | n.a. | Cognitive / Neurological (attentional deficits, cognitive impairment); Respiratory / Cardiac (shortness of breath) |
| P22 | PCcog (23) | m | 43 | 20.03.2020 | *15 | n.a. | n.a. | \| General/Systemic (Fatigue) \| \| --- \|  \|  \| \| --- \| |
| P23 | PCcog (24) | m | 54 | 23.02.21 | *5 | 0 | none | \| General/Systemic (Fatigue, Headache, Muscle/joint pain); Respiratory/Cardiac (Cough, Shortness of breath) \| \| --- \|  \|  \| \| --- \| |
| P24 | PCn (27) | m | 44 | 04/2020 | 3 | 2 | BioNTech, 17.05.21, 29.06.21 | General / Systemic (fatigue); Respiratory / Cardiac (cough, palpitations) |
| P25 | PCcog (25) | f | 35 | 03/2021 | *5,0 | n.a. | n.a. | Cognitive / Neurological (attentional deficits, cognitive impairment); Sensory (loss of smell/taste); General / Systemic (fatigue); Respiratory / Cardiac (shortness of breath); Psychiatric / Neuropsychiatric (sleep disturbances) |
| P26 | PCn (29) | m | 20 | n.a. | n.a. | n.a. | n.a. | General / Systemic (fatigue, sweating, cold intolerance); Respiratory / Cardiac (shortness of breath); Sensory (loss of smell/taste) |
| P27 | PCn (28) | f | 46 | 07.12.2020 | *9,5 | 3 | BioNTech 02.04.2021, 24.04.2021, 25.11.2021 | \| General/Systemic (Fatigue, Headache, Muscle/joint pain); Cognitive/Neurological (Cognitive impairment, Decreased responsiveness); Respiratory/Cardiac (Cough, Shortness of breath) \| \| --- \|  \|  \| \| --- \| |
| P28 | PCn (28) | f | 25 | 22.12.2020 | *9 | n.a. | n.a. | General / Systemic (fatigue, headaches); Psychiatric / Neuropsychiatric (sleep disturbances); Cognitive / Neurological (attentional deficits) |
| P29 | PCcog (24) | m | 79 | 01/2021 | *8,5 | 1 | BioNTech 16.06.2021 | General/Systemic (Fatigue); Respiratory/Cardiac (Shortness of breath, cough); Cognitive/Neurological (Cognitive impairment);  Psychiatric/Neuropsychiatric (Sleep disturbances) |
| P30 | PCn (30) | f | 41 | 18.12.2020 | *9,5 | 1 | BioNTech 12.05.2021 | \| General/Systemic (Fatigue, Headache, Muscle/joint pain, Dizziness, Decreased appetite); Psychiatric/Neuropsychiatric (Sleep disturbance); Cognitive/Neurological (Cognitive impairment); Respiratory/Cardiac (Cough, Shortness of breath) \| \| --- \|  \|  \| \| --- \| |
| P31 | PCn (26) | m | 36 | 02/2021 | *8 | 1 | BioNTech 04.06.2021 | \| General/Systemic (Fatigue); Psychiatric/Neuropsychiatric (Sleep disturbance); Cognitive/Neurological (Cognitive impairment); Respiratory/Cardiac (Cough, Shortness of breath) \| \| --- \|  \|  \| \| --- \| |
| P32 | PCn (28) | f | 50 | 12/2020 | *10,5 | 1 | BioNTech 24.04.2021 | \| General/Systemic (Fatigue, Headache, Muscle/joint pain); Psychiatric/Neuropsychiatric (Depressed mood, Anxiety, Sleep disturbance); Cognitive/Neurological (Cognitive impairment, Word retrieval difficulties  , attentional deficits); Respiratory/Cardiac (Cough, Shortness of breath); Sensory (Loss of smell/taste) \| \| --- \|  \|  \| \| --- \| |
| P33 | PCcog (24) | f | 34 | 03/2020-04/2020 | 1 | 2 | BioNTech 30.03.2021, 20.04.2021 | \| General/Systemic (Fatigue, Headache, Muscle/joint pain); Psychiatric/Neuropsychiatric (Depressed mood, Sleep disturbance); Cognitive/Neurological (Cognitive impairment); Respiratory/Cardiac (Cough, Shortness of breath) \| \| --- \|  \|  \| \| --- \| |
| P34 | PCcog (25) | m | 30 | 15.11.2020 | *12 | 2 | BioNTech 03.05.2021, 14.06.2021 | \| General/Systemic (Fatigue, Headache, Muscle/joint pain); Cognitive/Neurological (Cognitive impairment, Neuropathy, Brain fog, Orthostatic tachycardia); Respiratory/Cardiac (Cough, Shortness of breath) \| \| --- \|  \|  \| \| --- \| |
| P35 | PCn (26) | f | 54 | 26.04.2020 | *18,5 | 2 | AstraZeneca 17.02.2021, BioNTech 22.09.2021 | \| General/Systemic (Headache, Muscle/joint pain); Sensory (Loss of smell/taste) \| \| --- \|  \|  \| \| --- \| |
| P36 | PCn (26) | f | 43 | 29.10.2020 | *13 | n.a. | n.a. | \| General/Systemic (Fatigue, Headache, Muscle/joint pain); Psychiatric/Neuropsychiatric (Depressed mood, Sleep disturbance); Cognitive/Neurological (Cognitive impairment); Respiratory/Cardiac (Cough, Shortness of breath) \| \| --- \|  \|  \| \| --- \| |
| P37 | PCn (26) | m | 49 | 14.12.2020 | *11,5 | 1 | 15.06.2021 Jonson & Jonson | General/ systemic (fatigue) |
| P38 | PCn (26) | m | 24 | 17.12.2020 | *11 | 2 | BioNTech 28.05.2021; 09.07.2021 | \| Psychiatric/Neuropsychiatric (Depressed mood, Anxiety); Respiratory/Cardiac (Cough, Shortness of breath) \| \| --- \|  \|  \| \| --- \| |
| P39 | PCn (28) | m | 42 | 04/2021 | *7,5 | 1 | 21.04.2021 Jonson & Jonson | \| General/Systemic (Fatigue, Headache, Muscle/joint pain); Psychiatric/Neuropsychiatric (Sleep disturbance); Cognitive/Neurological (Cognitive impairment) \| \| --- \|  \|  \| \| --- \| |
| P40 | PCcog (20) | f | 44 | 10.12.2020 | *12 | 1 | 10.09.2021 | General/Systemic (fatigue, headache, muscle/joint pain); Psychiatric/Neuropsychiatric (sleep disturbances); Cognitive/Neurological (cognitive impairment, neuropathy); Respiratory/Cardiac (cough, shortness of breath); Sensory (loss of smell/taste) |
| P41 | PCn (26) | f | 48 | 24.12.2020 | *12 | 1 | 21.07.2021 BioNTech | \| General/Systemic (Fatigue); Psychiatric/Neuropsychiatric (Depressed mood); Cognitive/Neurological (Cognitive impairment); Sensory (Visual impairment) \| \| --- \|  \|  \| \| --- \| |
| P42 | PCn (30) | f | 37 | 28.12.2020 | *13,5 | 2 | 27.05.2021, 25.11.2021 BionTech | \| General/Systemic (Fatigue, Headache, Muscle/joint pain); Psychiatric/Neuropsychiatric (Sleep disturbance); Cognitive/Neurological (Cognitive impairment) \| \| --- \|  \|  \| \| --- \| |
| P43 | PCn (26) | m | 24 | 02/2021 | *11 | 2 | 09.06.2021, 06.07.2021 | \| General/Systemic (Fatigue); Cognitive/Neurological (Cognitive impairment); Respiratory/Cardiac (Cough, Shortness of breath); Sensory (Loss of smell/taste) \| \| --- \|  \|  \| \| --- \| |
| P44 | PCn (26) | f | 26 | 18.12.2020 | *13,5 | 2 | Biontech 04.08.21, 22.09.21 | \| General/Systemic (Fatigue, Headache, Muscle/joint pain); Cognitive/Neurological (Cognitive impairment, Neuropathy); Respiratory/Cardiac (Cough, Shortness of breath) \| \| --- \|  \|  \| \| --- \| |
| P45 | PCn (29) | f | 37 | 12.04.2021 | *9,5 | 1 | 11.09.2021 Biontech | \| General/Systemic (Fatigue, Headache, Muscle/joint pain); Psychiatric/Neuropsychiatric (Anxiety); Cognitive/Neurological (Cognitive impairment); Respiratory/Cardiac (Cough, Shortness of breath); Sensory (Loss of smell/taste) \| \| --- \|  \|  \| \| --- \| |
| P46 | PCcog (25) | f | 31 | 28.11.2020 | *14 | 2 | 02.06.2021, 06.12.2021 Biontech | \| General/Systemic (Fatigue, Headache, Muscle/joint pain); Psychiatric/Neuropsychiatric (Depressed mood, Sleep disturbance); Cognitive/Neurological (Cognitive impairment, Neuropathy); Respiratory/Cardiac (Cough, Shortness of breath); Sensory (Loss of smell/taste) \| \| --- \|  \|  \| \| --- \| |
| P47 | PCcog (25) | f | 43 | 26.04.2021 | *9,5 | 2 | 19.10.2021, 25.01.2022 Biontech | \| General/Systemic (Fatigue, Headache, Muscle/joint pain); Psychiatric/Neuropsychiatric (Depressed mood, Sleep disturbance); Cognitive/Neurological (Cognitive impairment) \| \| --- \|  \|  \| \| --- \| |
| P48 | PCcog (25) | f | 25 | 09.03.2021 | *11 | 2 | 05.10.2021, 26.01.2021 Biontech | n.a. |
| P49 | PCcog (25) | f | 73 | 02.04.2021 | *11 | 2 | 04.08.2021 Biontech, 12.01.2022 Moderna | \| General/Systemic (Fatigue, Headache, Muscle/joint pain); Psychiatric/Neuropsychiatric (Anxiety); Cognitive/Neurological (Cognitive impairment, Neuropathy); Respiratory/Cardiac (Cough, Shortness of breath) \| \| --- \|  \|  \| \| --- \| |
| P50 | PCcog (25) | F | 29 | 24.11.2021 | *3,5 | 1 | 16.12.2021 BionTech | General/Systemic (Fatigue, headache, Muscle/joint pain; Cognitive/Neurological (cognitive impairment); Sensory (Loss of smell/taste); Respiratory/ Cardiac (cough, shortness of breath) |
| P51 | PCn (29) | m | 32 | 02.04.2021 | *11,5 | 2 | 21.09.2021, 19.01.2022 BionTech | \| General/Systemic (Fatigue, Headache, Muscle/joint pain); Psychiatric/Neuropsychiatric (Sleep disturbance) \| \| --- \|  \|  \| \| --- \| |
| P52 | PCcog (22) | f | 58 | 07.05.2021 | *10,5 | 1 | 14.12.2021 Moderna | \| General/Systemic (Fatigue, Headache, Muscle/joint pain); Psychiatric/Neuropsychiatric (Anxiety, Sleep disturbance); Cognitive/Neurological (Cognitive impairment, Neuropathy); Sensory (Tinnitus) \| \| --- \|  \|  \| \| --- \| |
| P53 | PCcog (25) | f | 52 | 16.03.2021 | *12 | 2 | 11.08.2021, 21.12.2021 BionTech | \| General/Systemic (Fatigue, Headache, Muscle/joint pain); Psychiatric/Neuropsychiatric (Sleep disturbance); Cognitive/Neurological (Cognitive impairment); Respiratory/Cardiac (Cough, Shortness of breath) \| \| --- \|  \|  \| \| --- \| |
| P54 | PCn (28) | f | 21 | 11.02.2021 | *13,5 | 2 | 01.08.2021 Johnson&Johnson, 04.01.2022 BionTech | \| Cognitive/Neurological (attentional deficits) \| \| --- \|  \|  \| \| --- \| |
| P55 | PCn (26) | f | 46 | 08.12.2020 | *15,5 | 2 | 25.05.2021, 17.12.2021 BionTech | \| Cognitive/Neurological (Cognitive impairment); Sensory (Loss of smell/taste) \| \| --- \|  \|  \| \| --- \| |
| P56 | PCn (28) | f | 50 | 02/2021 | *13,5 | 2 | 22.07.2021, 04.01.2022 Comirnaty | \| General/Systemic (Fatigue, Headache, Muscle/joint pain); Psychiatric/Neuropsychiatric (Depressed mood); Cognitive/Neurological (Cognitive impairment) \| \| --- \|  \|  \| \| --- \| |
| P57 | PCn (27) | f | 41 | 15.01.2021 | *14,5 | 2 | 10.05.2021, 15.11.2021 BionTech | \| General/Systemic (Fatigue, Headache, Muscle/joint pain); Respiratory/Cardiac (Cough, Shortness of breath) \| \| --- \|  \|  \| \| --- \| |
| P58 | PCcog (23) | f | 63 | 15.03.2020 | *25 | 3 | 08.04.2021, 29.04.2021 BionTech; 16.12.2021 Moderna | \| General/Systemic (Fatigue, Headache, Muscle/joint pain); Psychiatric/Neuropsychiatric (Depressed mood, Anxiety, Sleep disturbance); Cognitive/Neurological (Cognitive impairment, Word retrieval difficulties, attentional deficits); Respiratory/Cardiac (Cough, Shortness of breath); Sensory (Loss of smell/taste) \| \| --- \|  \|  \| \| --- \| |
| P59 | PCcog (22) | f | 63 | 08.05.2021 | *11,5 | 3 | 06.01.2021, 28.01.2021, 11.11.2021 BionTech | \| General/Systemic (Fatigue, Headache, Muscle/joint pain); Psychiatric/Neuropsychiatric (Depressed mood, Anxiety, Sleep disturbance); Cognitive/Neurological (Cognitive impairment) \| \| --- \|  \|  \| \| --- \| |
| P60 | PCn (28) | m | 61 | 04/2021-02/2022 | 10 | 3 | 26.06.2021, 17.07.2021 BionTech, 21.12.2021 Moderna | \| Sensory (Loss of smell/taste) \| \| --- \|  \|  \| \| --- \| |
| P61 | PCcog (22) | m | 55 | 12/2020 | *16,5 | 2 | 20.02.2021, 10.11.2021 BionTech | \| General/Systemic (Fatigue, Headache, Muscle/joint pain); Psychiatric/Neuropsychiatric (Depressed mood); Cognitive/Neurological (Cognitive impairment, Neuropathy); Sensory (Loss of smell/taste) \| \| --- \|  \|  \| \| --- \| |
| P62 | PCcog (21) | m | 58 | 23.04.2021 | *12,5 | 2 | 02.09.2021, 24.02.2022 BionTech | \| General/Systemic (Fatigue, Headache, Muscle/joint pain); Psychiatric/Neuropsychiatric (Depressed mood, Sleep disturbance); Cognitive/Neurological (Cognitive impairment); Respiratory/Cardiac (Cough, Shortness of breath) \| \| --- \|  \|  \| \| --- \| |
| P63 | PCn (26) | m | 24 | 01/2021 | *17 | 2 | 09.06.2021, 06.07.2021 BionTech | \| General/Systemic (Fatigue); Psychiatric/Neuropsychiatric (Sleep disturbance); Cognitive/Neurological (Cognitive impairment); Respiratory/Cardiac (Cough, Shortness of breath) \| \| --- \|  \|  \| \| --- \| |
| P64 | PCcog (25) | m | 21 | n.a. | n.a. | n.a. | n.a. | n.a. |

n.a. = data not available; participants assessed in April/May 2021 were recruited at a time when large parts of the population had not yet been vaccinated; * indicates that long-COVID symptoms were still present at the time of data collection; for month-based symptom durations without a specific start date, calculations were standardized from the 15th of the respective month and rounded up to the nearest half or full month
